# Differential Roles of IDO1 and IDO2 in T and B Cell Inflammatory Immune Responses

**DOI:** 10.3389/fimmu.2020.01861

**Published:** 2020-08-18

**Authors:** Lauren M. F. Merlo, James B. DuHadaway, James D. Montgomery, Wei-Dan Peng, Peter J. Murray, George C. Prendergast, Andrew J. Caton, Alexander J. Muller, Laura Mandik-Nayak

**Affiliations:** ^1^Lankenau Institute for Medical Research, Wynnewood, PA, United States; ^2^Immunoregulation Group, Max Planck Institute of Biochemistry, Martinsried, Germany; ^3^Department of Pathology, Anatomy and Cell Biology, Sidney Kimmel Medical College, Thomas Jefferson University, Philadelphia, PA, United States; ^4^Sidney Kimmel Cancer Center, Thomas Jefferson University, Philadelphia, PA, United States; ^5^The Wistar Institute, Philadelphia, PA, United States

**Keywords:** experimental arthritis, influenza, immunization, B cells, murine model

## Abstract

Indoleamine-2,3-dioxygenase (IDO)1 and IDO2 are two closely related tryptophan catabolizing enzymes encoded by linked genes. The IDO pathway is also immunomodulatory, with IDO1 well-characterized as a mediator of tumor immune evasion. Due to its homology with IDO1, IDO2 has been proposed to have a similar immunoregulatory function. Indeed, IDO2, like IDO1, is necessary for the differentiation of regulatory T cells *in vitro*. However, compared to IDO1, *in vivo* studies demonstrated a contrasting role for IDO2, with experiments in preclinical models of autoimmune arthritis establishing a proinflammatory role for IDO2 in mediating B and T cell activation driving autoimmune disease. Given their potentially opposing roles in inflammatory responses, interpretation of results obtained using IDO1 or IDO2 single knockout mice could be complicated by the expression of the other enzyme. Here we use IDO1 and IDO2 single and double knockout (dko) mice to define the differential roles of IDO1 and IDO2 in B cell-mediated immune responses. Autoreactive T and B cell responses and severity of joint inflammation were decreased in IDO2 ko, but not IDO1 ko arthritic mice. Dko mice had a reduction in the number of autoantibody secreting cells and severity of arthritis: however, percentages of differentiated T cells and their associated cytokines were not reduced compared to IDO1 ko or wild-type mice. These data suggest that autoreactive B cell responses are mediated by IDO2, while autoreactive T cell responses are indirectly affected by IDO1 expression in the IDO2 ko mice. IDO2 also influenced antibody responses in models of influenza infection and immunization with T cell-independent type II antigens. Taken together, these studies provide evidence for the contrasting roles IDO1 and IDO2 play in immune responses, with IDO1 mediating T cell suppressive effects and IDO2 working directly in B cells as a proinflammatory mediator of B cell responses.

## Introduction

Indoleamine-2,3-dioxygenase (IDO)1 and IDO2 are two closely related tryptophan catabolizing enzymes induced under inflammatory conditions that contribute to immune responses. IDO1 is widely expressed in both immune and non-immune tissues, whereas expression of IDO2 is restricted to liver, kidney, and antigen presenting cells (dendritic cells and B cells) (1–3). Both IDO1 and IDO2, along with the unrelated enzyme tryptophan dioxygenase (TDO), catalyze the first and rate-limiting step in the catabolism of tryptophan to kynurenine (4). However, IDO2 has much weaker tryptophan catabolizing activity than IDO1, both as measured by enzyme activity *in vitro* and by analyzing levels of serum kynurenine in the absence of each enzyme *in vivo* (3, 5–8). Due to their homology, IDO1 and IDO2 had been thought to play redundant roles in immune responses; however, recent results from *in vivo* models of cancer and autoimmunity suggest that IDO2 may play a role in immune function distinct from IDO1 (6, 9). Understanding the contribution of IDO1 and IDO2 to immune responses is complicated by the fact that the genes encoding each enzyme are linked and likely arose by gene duplication (10).

IDO1 has been shown to inhibit T cell activation and induce T regulatory cell development *in vitro* (11, 12). *In vivo*, IDO1 is best known for its immunoregulatory role in mediating tumor immune evasion (13–15). Elevated IDO1 expression has been described in several human tumors and mouse tumor models (16–18) and IDO1 deficient mice are resistant to tumor formation in preclinical models (19). This inhibitory function of IDO1 is thought to primarily be through the induction of T regulatory cells, although recent studies have described a novel function for IDO1 in inflammatory neovascularization, that could be just as, if not more, important in some tumor settings (20). In contrast to its link to regulating immune responses in cancer, the effect of IDO1 on autoimmune responses has been less clear. Some studies describe a regulatory function (21–24), while others suggest a pro-inflammatory role (8, 25) or no role at all (1, 26, 27).

IDO2 has been much less studied than IDO1 and its role in immune function is still being determined. IDO2 was required for T regulatory cell activation under the same assay conditions that IDO1 was shown to be critical (8). However, IDO2's effect on tumor development is ambiguous, with data supporting both immunoregulatory (28) and proinflammatory roles (29) depending on the model used. Likewise, the requirement for IDO2 in normal immune function is not known. IDO2 does not appear to be necessary for overall immune function, as IDO2 deficient mice do not show gross defects in immune cell development or titers of total serum antibody (1, 8). In contrast, IDO2 has been shown to play a pro-inflammatory role in the development of B cell-mediated autoimmunity. This specific pathogenic function was first described in the KRN model of autoimmune arthritis (1) and later confirmed in the collagen induced arthritis model (30). Both autoreactive T and B cell responses were significantly reduced in arthritic mice lacking IDO2 (1) and B cells were identified as the critical IDO2-expressing cell mediating disease (31).

Taken together, IDO1 and IDO2 appear to play opposing roles in inflammatory immune responses, with IDO1 an important inhibitor of effector T cell-mediated responses, especially in the context of cancer, and IDO2 a critical proinflammatory mediator of B cell-mediated autoimmunity. As such, interpretation of results obtained using IDO1 or IDO2 single knockout (ko) mice could be complicated by the remaining expression of the counteracting enzyme. To address this issue, here we use mice in which both IDO1 and IDO2 have been deleted (double knockout, dko) to determine the relative contribution of IDO1 and IDO2 to B cell-mediated immune responses. Using the dko mice together with the KRN model of arthritis, we demonstrate that IDO2 mediates the autoreactive B cell response driving arthritis through an IDO1-independent mechanism. In contrast, the decreased autoreactive T cell response found in IDO2 ko mice was dependent upon IDO1 expression, highlighting the importance of using double ko mice to deconvolute IDO2's functional interrelationship with IDO1. To determine if IDO2 has the same impact on normal B cell responses as it does in autoreactive ones, we used IDO1 and IDO2 single and double ko mice together with well-characterized models of *in vitro* and *in vivo* B cell activation. IDO2 was not required in all models, but specifically mediated B cell antibody production in response to influenza infection and immunization with a T cell-independent type II model antigen. In these contexts, similar reduced responses were seen in IDO2 single and double ko mice, confirming that IDO2 mediates B cell activation in an IDO1-independent manner.

## Materials and Methods

### Mice

KRN TCR Tg (32), IDO1 deficient (IDO1 ko) (33) IDO2 ko (8), and IDO1/IDO2 double ko (dko) (34) mice on a C57BL/6 background have been described. Arthritic mice were generated by breeding KRN Tg C57BL/6 mice expressing the I-A^g7^ MHC Class II molecule (KRN.g7). This process was repeated to generate arthritic mice lacking IDO1, IDO2, or both IDO1 and IDO2 (IDO1 ko KRN.g7, IDO2 ko KRN.g7, or dko KRN.g7). KRN.g7 mice develop arthritis with similar kinetics as the original K/BxN mice (35). All mice were bred and housed under specific pathogen free conditions in the animal facility at the Lankenau Institute for Medical Research. Studies were performed in accordance with National Institutes of Health and Association for Assessment and Accreditation of Laboratory Animal Care guidelines with approval from the LIMR Institutional Animal Care and Use Committee.

### Arthritis Incidence

The two rear ankles of wt, IDO1, IDO2 ko, and dko KRN.g7 mice were measured starting at weaning (3 wk of age). Measurement of ankle thickness was made above the footpad axially across the ankle joint using a Fowler Metric Pocket Thickness Gauge. Ankle thickness was rounded off to the nearest 0.05 mm.

### ELISPOT Assay

Anti-GPI antibody secreting cells were measured by ELISpot as described (36). Briefly, cells from the joint draining lymph nodes (axillary, brachial, and popliteal LNs) from 6 week-old KRN.g7, IDO1 ko KRN.g7, IDO2 ko KRN.g7, and dko KRN.g7 mice were plated at 4 × 10^5^ cells per well and diluted serially 1:4 in Multiscreen HA mixed cellulose ester membrane plates (Millipore) coated with GPI-his (10 μg/ml). The cells were incubated on the Ag-coated plates for 4 h at 37°C. The Ig secreted by the plated cells was detected by Alkaline Phosphatase-conjugated goat anti-mouse total Ig secondary Ab (Southern Biotechnology Associates) and visualized using NBT/BCIP substrate (nitroblue tetrazolium/5-bromo-4-chloro-3-indolyl phosphate; Sigma).

### NP Immunization

Wild-type (wt), IDO1 ko, IDO2 ko, or dko C57BL/6 mice were immunized i.p. with 50 μg (4-Hydroxy-3-Nitrophenyl)Acetyl (NP)-Ficoll, 50 μg NP-LPS, or 100 μg NP-keyhole limpet hemocyanin (KLH), precipitated in alum (Biosearch Technologies). NP-KLH immunized mice were boosted 21 days after initial immunization with 100 μg NP-KLH precipitated in alum. NP-Ficoll and NP-LPS immunized mice were bled on day 7 and NP-KLH mice were bled on day 10 after primary and secondary immunizations. NP titers were measured by ELISA.

### Influenza Infection

Influenza virus PR8 (A/Puerto Rico/8/34) was grown and purified as described (37). Wt, IDO1 ko, IDO2 ko, or dko C57BL/6 mice were infected i.n. with 200 TCID50 influenza virus (A/PR/8/34) in 50 μl PBS. Cohorts of mice were bled on days 0, 5, 7, and 10. Titers of anti-PR8 Ig were measured by ELISA.

### *In vitro* Stimulation

Two spleens each from wt, IDO1 ko, IDO2 ko, or dko C57BL/6 mice were pooled and naïve B cells isolated by negative selection with anti-CD43 MACS beads (Miltenyi Biotec, purity >96%). Purified B cells were labeled with 5 μM CFSE (Fisher) for 10 min, then cultured with media alone, 0.2 μg/ml PAM3CSK4, 10 μg/ml Poly I:C, 10 μg/ml LPS, 100 μM Loxoribine, 1 μM CpG ODN 1826, 2 μg/ml anti-CD40 + 50 ng/ml IL-4, or 50 ng/mL PMA + 500 ng/ml ionomycin (Invivogen and Sigma). After 48 h, the cells were analyzed with antibodies to CD80 and CD86 (eBioscience), CD25 and CD138 (BioLegend), and CFSE staining by flow cytometry. The samples were acquired on a BD FACSCanto II flow cytometer using FACSDiva Software (BD Bioscience). Surface marker expression and division index were analyzed using FlowJo Software (TreeStar). Secreted Ig in the supernatant was measured by ELISA.

### ELISA Assays

Serum samples were plated at an initial dilution of 1:100 and diluted serially 1:4 on Immulon II plates coated with NP_4_-BSA (Biosearch Technologies) or purified PR8 virus, respectively. The serum titer was defined as the reciprocal of the last dilution that gave an O.D.>3x background. To measure Ig secretion *in vitro*, supernatants were plated undiluted and then diluted serially 1:5 on Immulon II plates coated with anti-mouse Ig_H+L_ (Jackson Immunoresearch). Ig concentration was determined by comparison to a standard curve of IgM (BD Bioscience). Goat anti-mouse IgM-HRP, IgG-HRP (Southern Biotechnology), and donkey anti-mouse IgG_H+L_-HRP (Total Ig, Jackson Immunoresearch) were used as secondary antibodies. Antibody was detected using ABTS substrate (Fisher).

### Immunohistochemistry

Spleens from NP-Ficoll and NP-LPS immunized mice were harvested on day 7 and NP-KLH immunized mice on day 10, embedded in OCT, frozen in liquid nitrogen cooled 2-methyl-butane, sectioned, and fixed with acetone. Spleen sections were stained with B220-biotin (BioLegend) and anti-Igλ-AP (Southern Biotech). Streptavidin-HRP (Southern Biotech) was used as a secondary antibody. AP and HRP were detected using Fast-Blue BB base and 3-amino-9-ethylcarbazole (Sigma), respectively. Sections were imaged using a Zeiss Axioplan microscope with a Zeiss Plan-Apochromat 10x/0.32 objective and Zeiss AxioCam HRC camera using AxioVision 4.7.1 software. The images were then processed using Adobe Photoshop CC software.

### Analysis of B Cell Subsets

Cells were harvested from the spleens of 8–10 week old wt, IDO1 ko, IDO2 ko, and dko ko C57BL/6 mice and stained with antibodies to CD21 and IgM (BD Bioscience), CD23 eBioscience), B220, and CD93 (BioLegend). The samples were acquired on a BD FACSCanto II flow cytometer using FACSDiva Software and analyzed using FlowJo Software.

### Analysis of T Helper Subsets

Joint draining LN cells from 6 week old KRN.g7, IDO1 ko KRN.g7, IDO2 ko KRN.g7, and dko KRN.g7 mice were harvested and stained for CD4^+^ T cells (BioLegend) and the following markers to distinguish T_H_ subsets: bcl6 (BD Bioscience), foxP3 (Biolegend), gata3, rorγt, T-bet (all from eBioscience) as described (1). The samples were acquired on a BD FACSCanto II flow cytometer using FACSDiva Software and analyzed using FlowJo Software.

### Intracellular IL-21

Cells from the joint draining LNs of 6 week old KRN.g7, IDO1 ko KRN.g7, IDO2 ko KRN.g7, and dko KRN.g7 mice were harvested and cultured for 4 h with 50 ng/ml PMA, 500 ng/ml ionomycin, and 3 μg/ml brefeldin A. After 4 h, cells were harvested, surface stained for CD4 (eBioscience), fixed and permeabilized (IC Fixation and Permeabilization Buffer, eBioscience), then stained for intracellular IL-21 (eBioscience). The samples were acquired on a BD FACSCanto II flow cytometer using FACSDiva software and analyzed with FlowJo software.

### Cytokine Secretion

Cells from the joint draining LNs of 6 week old KRN.g7, IDO1 ko KRN.g7, IDO2 ko KRN.g7, and dko KRN.g7 mice were harvested and cultured in with PMA (50 ng/ml) + ionomycin (500 ng/ml) for 24 h. The supernatants were then harvested and analyzed for the levels of IL-17, TNFα, and IFNγ by cytometric bead array (BD Biosciences). The samples were stained according to manufacturer instructions and analyzed on a BD FACSCanto II flow cytometer using FACSDiva software. Cytokine concentrations were calculated by comparing to standard curves using FACS array analysis software (BD Biosciences).

### Antigen Presenting Cell (APC) Assay

CD4^+^ T cells were purified by positive selection with anti-CD4 MACS beads (Miltenyi Biotec) from the spleen and lymph nodes of KRN tg B6 mice. 2 × 10^5^ KRN T cells were cultured with 1 × 10^5^ irradiated (2 Gy) splenocytes from wt, IDO1 ko, IDO2 ko, or dko C57BL/6.g7 mice at varying concentrations of GPI peptide (LSIALHVGFDHFE) in a final volume of 100 μL. After 68 h, 20 μL MTS assay reagent (Promega CellTiter96 AQueous One Solution Cell Proliferation Assay) was added and cultures read at A490 after 4 h (72 h total). To measure upregulation of activation markers, a 2:1 ratio of T cells:APCs were cultured with 3.16μM GPI peptide for 48 h and stained with anti-CD25 (BioLegend) and anti-CD69 (eBiosciences) by flow cytometry.

### Western Blotting

Liver and epididymis tissue were harvested from individual wt, IDO1 ko, IDO2 ko, and dko C57BL/6 mice. Spleens from C57BL/6 wt, IDO1 ko, IDO2 ko, or dko mice were harvested from 3 mice/genotype and pooled prior to purification. T cells were purified by MACS bead magnetic purification (Miltenyi Biotec) using CD90.2 microbeads for total T cell purification by positive selection and B cells were purified using CD43 microbeads beads (Miltenyi) by negative selection. T cell purity was >93% and B cell purity >96%. Naïve cells were used immediately. Activated T cells were stimulated 48 h with 5 μg/mL plate-bound αCD3 (clone 145-2C11, BioLegend) and 2 μg/ml soluble αCD28 (clone 37.51, BioLegend). Activated B cells were stimulated 48 h with 25μg/ml LPS (Sigma-Aldrich) + 50 ng/mL IL-4 (BioLegend). Liver, epididymis, and naïve and activated B/T cells were homogenized with a Beadbug microtube homogenizer (Sigma) in the presence of RIPA buffer containing protease and phosphatase inhibitors. Cell and tissue lysates were centrifuged and protein concentrations determined. Equal protein per sample (30 μg/lane) was used for liver and epididymis. Due to the low expression level of IDO1/2 in lymphocytes, the maximum protein obtained (naïve cells: 25–36 μg; activated cells: 60–80 μg) was used to help visualize any potential expression. Protein was fractioned using standard SDS-PAGE and blotted to Immobilon-NC membranes (Millipore, USA). After blocking, blots were incubated at 4°C overnight with primary antibody, either conjugated directly to HRP, or followed by incubation with an HRP-conjugated secondary antibody. Blots were developed with HYGLO Quickspray chemiluminescent HRP reagent (Denville Scientific) and analyzed using a ChemiDoc System with Image Lab Software (Biorad). Primary antibodies to the following antigens were used: IDO1 (Millipore Sigma); IDO2 (Santa Cruz); and GAPDH (Invitrogen). HRP-conjugated anti-mouse Igκ (Jackson Immunoresearch) was used as a secondary antibody to detect IDO1 and GAPDH.

### IDO1 and IDO2 RNA Expression

Liver and epididymis tissue were harvested from individual wt, IDO1 ko, IDO2 ko, and dko C57BL/6 mice. Spleens from C57BL/6 wt, IDO1 ko, IDO2 ko, or dko mice were harvested from 3 mice/genotype and pooled prior to purification. T cells were purified by MACS bead magnetic purification (Miltenyi Biotec) using CD90.2 microbeads for total T cell purification by positive selection and B cells were purified using CD43 microbeads beads (Miltenyi) by negative selection. T cell purity was >93% and B cell purity >96%. Cells were cultured in media alone (unstimulated), 5 μg/mL plate-bound αCD3 + 2 μg/ml soluble αCD28 (T cells), or 25 μg/ml LPS (Sigma-Aldrich) + 50 ng/mL IL-4 (B cells). After 48 h, cells were harvested, RNA was extracted with the RNEasy mini kit (Qiagen), and first strand cDNA synthesized using oligo-dT primer (Promega GoScript). IDO1 and IDO2 expression were measured by real time PCR using SYBR Green (Sigma-Aldrich). Expression of target gene IDO2 was determined relative to β-2-microglobulin (β2M) and calculated as 2∧^−^(Ct_Targetgene_-Ct_b2M_) as primers had similar efficiencies. Primers: IDO1, 5′-CCCACACTGAGCACGGACGG-3′ and 5′-TTGCGGGGCAGCACCTTTCG-3′, IDO2, 5′-CAATCCAGCCATGCCTGTGGGG-3′ and 5′-TGGGCTGCACTTCCTCCAGAGT-3′, and β2M 5′-CTCGGTGACCCTGGTCTTTC-3′ and 5′-TTGAGGGGTTTTCTGGATAGCA-3′.

### Kynurenine Assay

Serum and spleens were harvested from C57BL/6 wt, IDO1 ko, IDO2 ko, or dko mice Spleens were pooled from 3 mice/genotype prior to purification. T cells were purified by MACS bead magnetic purification (Miltenyi Biotec) using CD90.2 microbeads for total T cell purification by positive selection and B cells were purified using CD43 microbeads beads (Miltenyi) by negative selection. T cell purity was >93% and B cell purity >96%. Cells were cultured for 48 h in media alone (unstimulated), 5 μg/mL plate-bound αCD3 + 2 μg/ml soluble αCD28 (T cells), or 25 μg/ml LPS (Sigma-Aldrich) + 50 ng/mL IL-4 (B cells). Human 293-T-REx™ cells stably transfected with murine IDO1, IDO2, or untransfected controls (parental) under the control of the Tet repressor were used as positive controls for enzyme activity. IDO1 and IDO2 expression in 293-T-REx™ cells was induced with 2 μg/ml doxycycline for 48 h. Serum and harvested supernatants were analyzed for kynurenine levels using the IDK high sensitivity Kynurenine ELISA kit according to manufacturers instructions (Immunodiagnostik). Kynurenine levels were calculated by comparison to a standard curve.

### Statistical Analysis

Statistical significance was determined using one or two way-ANOVA followed by comparison of means with Tukey's *post-hoc* multiple comparison correction, an unpaired Student's *t*-test, or the Mann-Whitney non-parametric test using GraphPad Prism Software (GraphPad Software, Inc).

## Results

### IDO2 Expression Is Reduced in IDO1 ko Mice

**Figure 1:**
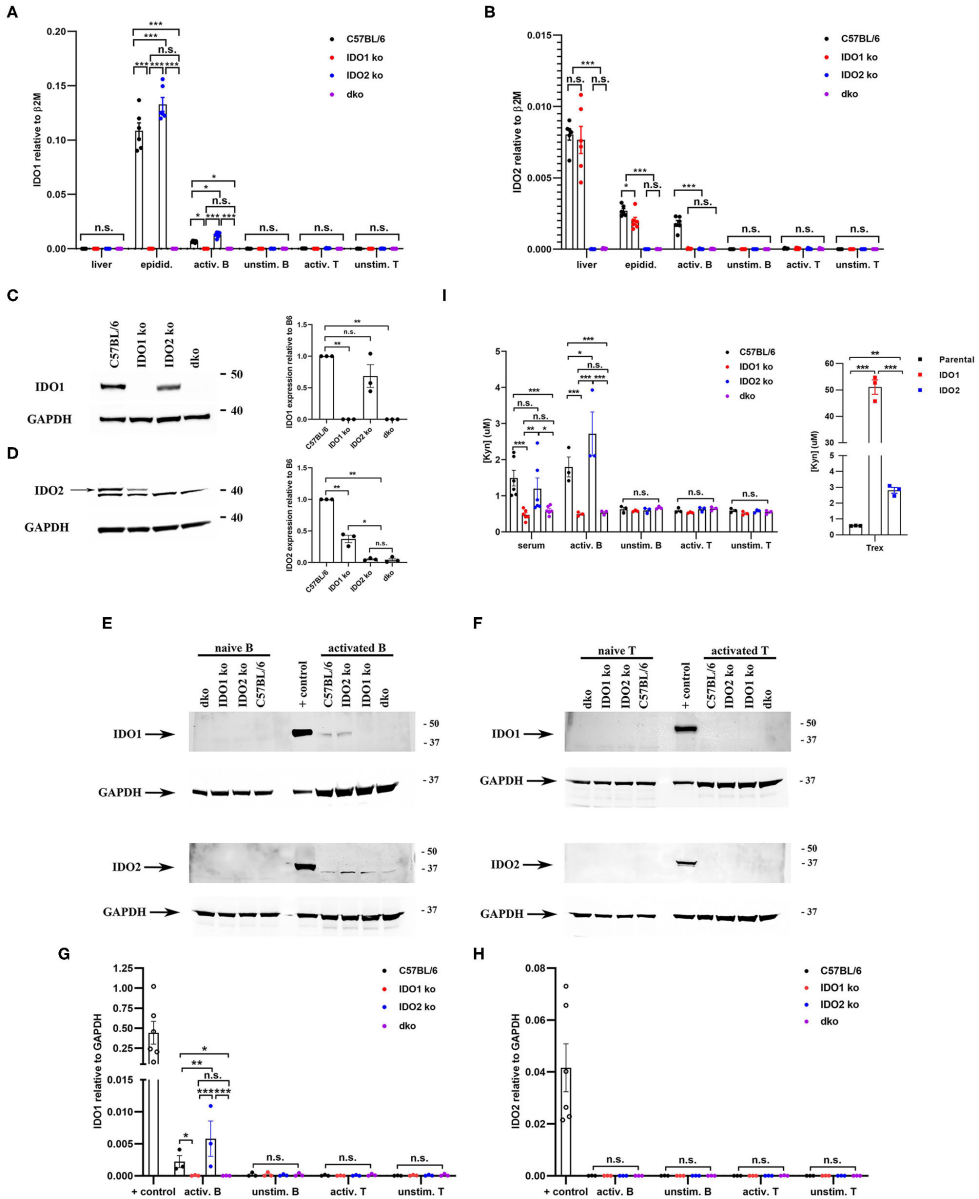
Differential expression of IDO1 and IDO2 in knockout mice. Purified B and T cells were cultured for 48 h either unstimulated (unstim.) or activated (activ.) with LPS + IL-4 (B cells) or anti-CD3 + anti-CD28 (T cells). **(A)** IDO1 and **(B)** IDO2 mRNA expression in liver, epididymis, and unstimulated/activated B and T cells isolated from C57BL/6, IDO1 ko, IDO2 ko, and dko mice was measured by qRT-PCR. Symbols represent individual pools of 3 mice and histograms show the mean ± SEM of IDO1 and IDO2 relative to β2M for *n* = 6 pools/genotype. **(C,D)** Protein lysates from **(C)** epididymis, **(D)** liver, **(E)** unstimulated/activated B cells, and **(F)** unstimulated/activated T cells from IDO1 ko, IDO2 ko, dko, or wt C57BL/6 mice were immunoblotted with antibodies to IDO1 and IDO2. Blots were then probed with anti-GAPDH as a loading control. Representative blot of 3 total. Molecular weights are indicated. The IDO2-specific band is indicated with an arrow. Symbols represent individual mice and histograms show the mean ± SEM ratio of **(G)** IDO1 or **(H)** IDO2, normalized to GAPDH, relative to the C57BL/6 control for *n* = 3 blots. **(I)** Serum and unstimulated/activated B and T cells supernatants (48 h) were analyzed for kynurenine (Kyn) by ELISA. 293-T-REx™ cells stably transfected with murine IDO1, IDO2, or untransfected controls (parental) were used as positive controls for enzyme activity. Symbols respresent individual mice (serum) or pools of 3 mice (B and T cells) and histograms show the mean ± SEM for *n* = 6/genotype (serum) and *n* = 3/genotype (B and T cells). *P*-values were calculated by two-way ANOVA with *post-hoc* testing by Fisher's LSD test. ^*^p < 0.05, ^**^p < 0.01, ^***^p < 0.001, n.s., not significant.

KRN.g7 mice develop a joint-specific inflammatory autoimmune response mediated by autoantibodies to the glycolytic enzyme glucose-6-phosphate isomerase (38–40). Using this model, we previously demonstrated reduced autoantibody production and a significantly attenuated course of disease in IDO2 ko, but not IDO1 ko, KRN.g7 mice (1). This suggests that IDO2 plays a fundamentally different role than IDO1 in mediating inflammatory immune responses. However, the reduced arthritis in IDO2 ko mice could be due to alterations in the expression or activity of IDO1 in the mice. Because of the conserved physical proximity of the IDO1 and IDO2 genes, it has been proposed that deletion of one gene could affect the expression of the other (41). In wt C57BL/6 mice, IDO1 mRNA is expressed at high levels in the epididymis and low levels in activated B cells (Figure 1A), whereas IDO2 is expressed at high levels in the liver and low levels in the epididymis and activated B cells (Figure 1B). Overall IDO2 expression relative to the housekeeping gene was much lower than IDO1 (Figures 1A,B). IDO1 is not expressed in the liver and neither IDO1 nor IDO2 mRNA were detectable in unstimulated B cells or unstimulated or activated T cells (Figures 1A,B). To determine if IDO1 deletion altered the expression of IDO2 or if IDO2 deletion affected the expression of IDO1, IDO1 and IDO2 mRNA and protein were measured in IDO1 ko, IDO2 ko, and dko mice. At the mRNA level, IDO1 ko mice have reduced levels of IDO2 in the epididymis and activated B cells, but not in the liver, potentially due to an increase in alternatively spliced transcripts in certain tissues [Figure 1B and refs. (1, 8)]. In contrast, IDO1 mRNA levels were elevated in the epididymis and activated B cells in IDO2 ko mice (Figure 1A).

**Figure 2:**
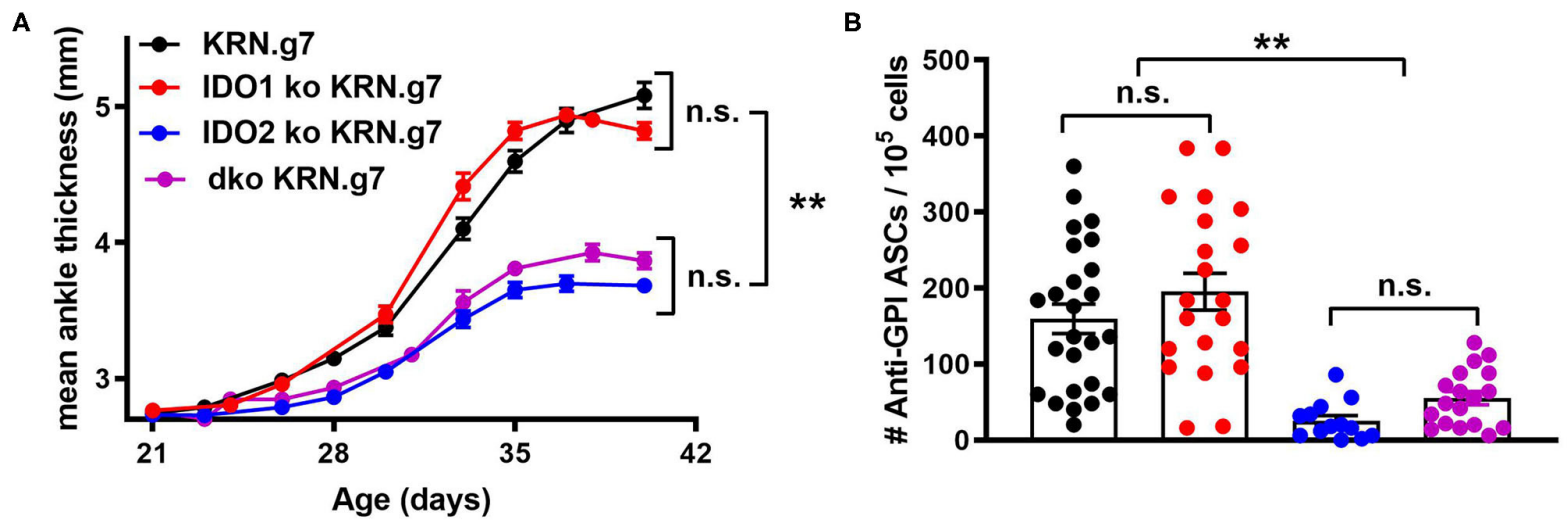
Deletion of both IDO1 and IDO2 inhibits autoantibody production and alleviates arthritis, phenocopying IDO2 single knockout mice. **(A)** Rear ankles were measured as an indication of arthritis and represented as mean ankle thickness ± SEM from *n* = 14 wt, *n* = 10 IDO1 ko, *n* = 12 IDO2 ko, and *n* = 9 IDO1/IDO2 (dko) KRN.g7 mice. **(B)** The number of anti-GPI ASCs from the joint draining lymph node was determined using an ELISpot assay. Symbols represent individual mice and histograms show the mean number of ASC ± SEM from *n* = 25 wt, *n* = 21 IDO1 ko, *n* = 13 IDO2 ko, and *n* = 18 dko KRN.g7 mice, pooled from a minimum of 4 independent litters of each genotype. *P*-values were calculated by one way-ANOVA followed by comparison of means with Tukey's *post-hoc* multiple comparison correction. ^**^p < 0.01, n.s., not significant.

To determine if these changes in mRNA were also present at the protein level, we first measured IDO1 and IDO2 in the tissues with the highest mRNA expression of IDO1 (epididymis) and IDO2 (liver) (Figures 1C,D and Supplementary Figure 1). In the epididymis, IDO1 protein is expressed in IDO2 ko mice at similar levels to that in wt mice. In contrast, IDO2 levels in the liver are decreased in IDO1 ko mice to about 40% of the level in wt mice. To determine if differences in IDO1/IDO2 protein were also found in lymphocytes, we measured IDO1 and IDO2 protein levels in unstimulated and activated B and T cells (Figures 1E–H and Supplementary Figure 2). IDO1 protein is expressed at higher levels in activated B cells from IDO2 ko mice compared to C57BL/6 mice, similar to its elevation at the RNA level (Figures 1E,G). Although IDO2 mRNA is clearly present in activated B cells from C57BL/6 mice, the level of IDO2 protein was below the level of detection by Western blotting (Figures 1E,H). Consistent with the mRNA results, neither IDO1 nor IDO2 protein is detectable in unstimulated B cells or unstimulated or activated T cells (Figures 1E–H). As expected, control IDO1 ko and IDO2 ko mice lack IDO1 and IDO2, respectively, and dko mice lack both IDO1 and IDO2 protein in all tissues/cell types tested (Figures 1C–H). These data demonstrate that deletion of one IDO gene affects the expression of the other. IDO1 ko mice have reduced levels of IDO2, whereas IDO2 ko mice have increased levels of IDO1 in some tissues/cell types. Importantly, the reduced level of IDO2 in IDO1 ko mice was still sufficient to drive arthritis in the KRN model, as no differences were seen between wt and IDO1 ko KRN.g7 mice in arthritis onset or severity [Figure 2A and ref. (1)].

Functionally, IDO1 and IDO2 are best known as tryptophan catabolizing enzymes, responsible for the first and rate-limiting step in the catabolism of tryptophan to kynurenine (4). To assess enzymatic activity, kynurenine levels were measured in the serum and culture supernatants from unstimulated and activated B and T cells from wt, IDO1 ko, IDO2 ko, and dko C57BL/6 mice (Figure 1I). Human 293-T-REx™ cells stably transfected with murine IDO1, IDO2, or untransfected controls (parental) under the control of the Tet repressor were used as positive controls for enzyme activity. Higher levels of kynurenine were produced by IDO1 (51.09 ± 2.7 μM) than IDO2 (2.812 ± 0.2 μM) expressing T-REx™ cells, consistent with previous reports demonstrating that IDO1 has much stronger enzymatic activity than IDO2 (3, 5–8). Supernatants from unstimulated B cells and unstimulated or activated T cells did not have levels of kynurenine above background. Kynurenine was elevated in the serum and activated B cell supernatant from wt and IDO2 ko, but not IDO1 ko or dko mice, suggesting that the kynurenine was due to IDO1, but not IDO2, enzymatic activity (Figure 1I).

### Joint Inflammation and Autoantibody Production Are Reduced in IDO1/IDO2 Double ko Mice

To determine if eliminating IDO1 would reverse the effect of IDO2 deficiency, the KRN.g7 model was bred onto an IDO1/IDO2 double knockout (dko) background and compared to IDO1 ko, IDO2 ko, or IDO wt KRN.g7 mice. Disease was monitored in the mice by measuring swelling in the rear ankles as an indication of arthritis. KRN.g7 mice begin to develop arthritis starting around 4 weeks of age, with peak inflammation between 5 and 6 weeks of age. As shown previously, deletion of IDO2, but not IDO1, delayed the time of onset and decreased the overall severity of joint inflammation [Figure 2A and ref. (1)]. Arthritis in dko KRN.g7 mice was indistinguishable from that in IDO2 ko KRN.g7 mice (Figure 2A). At 6 weeks of age, the number of autoantibody secreting cells in the joint draining lymph nodes was quantified by ELISpot. Autoantibody secreting cells were prevalent in the joint draining LNs of wt and IDO1 ko KRN.g7 mice, whereas they were significantly decreased in IDO2 ko mice. Autoantibody secreting cells were also decreased in dko KRN.g7 mice, consistent with their overall reduced severity of arthritis (Figure 2B). This indicates that absence of IDO2 alone, independent of the presence or absence of IDO1 expression, was responsible for the reduced autoreactive B cell response and alleviation of arthritis in the IDO2 ko mice.

### Decreased Autoreactive T Cell Activation in IDO2 ko Mice Is Mediated by IDO1

**Figure 3:**
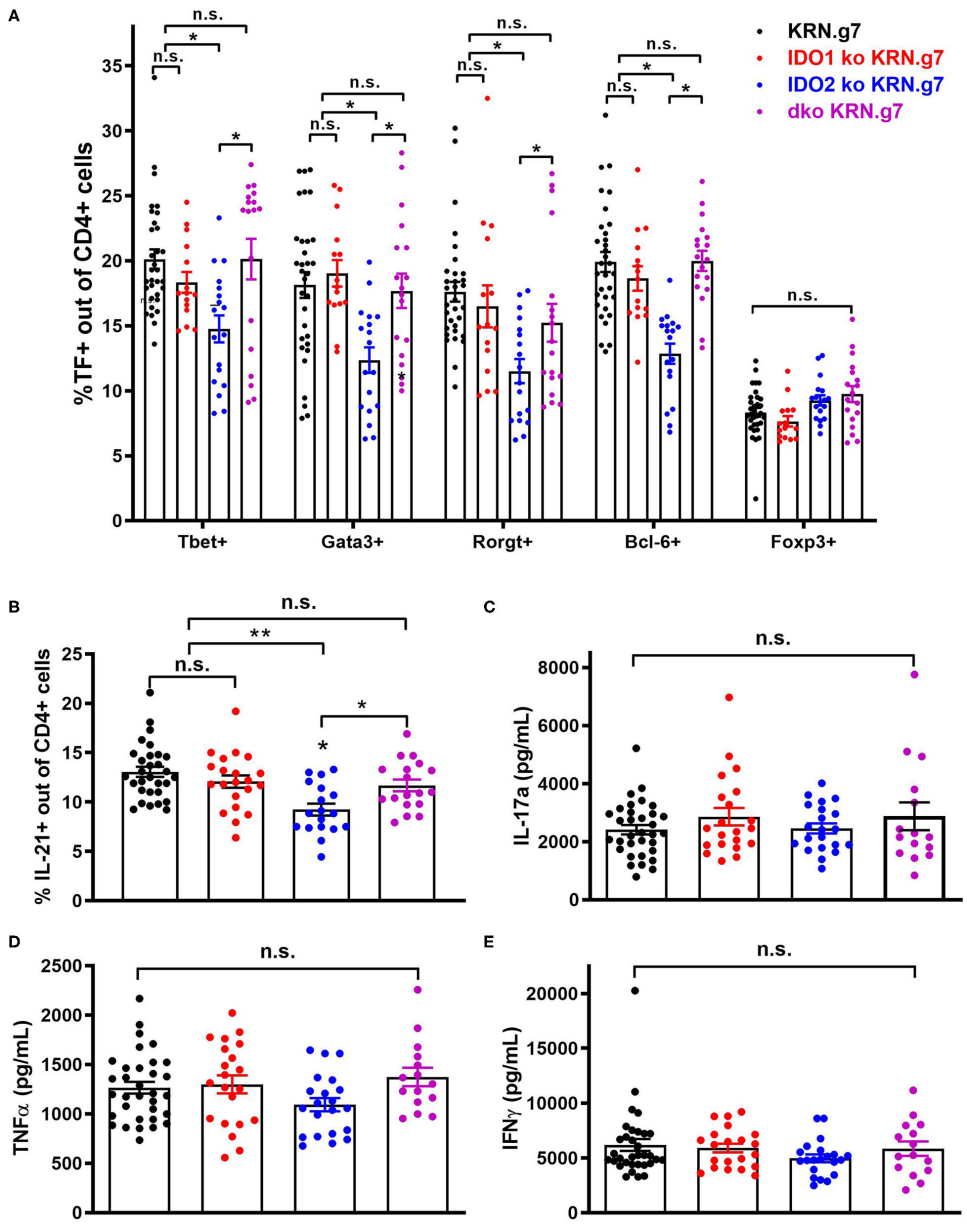
Decreased autoreactive T cell activation in IDO2 ko mice is mediated by IDO1. **(A)** Frequency of CD4^+^ T helper cell subpopulations were measured by flow cytometry by intracellular staining for the transcription factors T-bet (Th1), GATA-3 (Th2), RORγt (Th17), Bcl-6 (Tfh), and FoxP3 (Treg). Symbols represent individual mice and histograms show mean % ± SEM of each subpopulation out of total CD4^+^ T cells. **(B)** Cells from the joint dLNs were cultured for 4 h in PMA + ionomycin + brefeldin A. Intracellular IL-21 was measured by flow cytometry. Symbols represent individual mice and histograms show mean % IL-21^+^ cells ± SEM out of total CD4^+^ T cells. *N* = 32 wt, *n* = 21 IDO1 ko, *n* = 18 IDO2 ko, and *n* = 18 dko KRN.g7 mice, pooled from 9 independent experiments. **(C–E)** Cells from the joint dLNs were cultured for 24 h in PMA + Ionomycin. **(C)** IL-17a, **(D)** TNFα, and **(E)** IFNγ were measured in the supernatants by cytometric bead array. Symbols represent individual mice and histograms show mean concentration ± SEM from *n* = 33 wt, *n* = 21 IDO1 ko, *n* = 21 IDO2 ko, and *n* = 15 dko KRN.g7 mice, pooled from 9 independent experiments. *P*-values were calculated by one way-ANOVA followed by comparison of means with Tukey's *post-hoc* multiple comparison correction. ^*^p < 0.05, ^**^p < 0.01, n.s., not significant.

**Figure 4:**
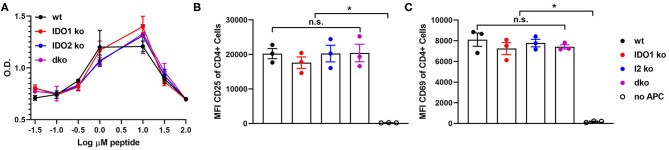
Antigen presenting function is not affected by deletion of IDO1 or IDO2. Naive KRN T cells were cultured with irradiated splenocytes from wt, IDO1 ko, IDO2 ko, or dko C57BL/6.g7 mice and GPI peptide. **(A)** Proliferation was measured by the MTS assay at 72 h. Graph is from a representative experiment of 3 total showing mean O.D. ± SD from 3 replicate wells. **(B,C)** Upregulation of the activation markers CD25 and CD69 were measured by flow cytometry at 48 h. Symbols represent individual mice and histograms show mean fluorescence intensity (MFI) ± SEM from 3 individual experiments. *P*-values were calculated by one way-ANOVA followed by comparison of means with Tukey's *post-hoc* multiple comparison correction. ^*^p < 0.05, n.s., not significant.

In addition to diminished autoreactive B cell responses and joint inflammation, IDO2 ko KRN.g7 mice had an overall reduction in autoreactive T cell responses (1). In particular, percentages of CD4^+^ T cells expressing the T helper (Th) transcription factors Tbet (Th1), Gata-3 (Th2), Rorγt (Th17), and Bcl-6 (Tfh) were consistently lower in IDO2 ko compared to wt KRN.g7 mice. Likewise, the percentage of IL-21^+^ CD4^+^ T cells was also reduced [ref. (1) and Figures 3A,B and Supplementary Figure 3], whereas levels of inflammatory cytokines IL-17a, TNFα, and IFNγ were similar to wt KRN.g7 mice [ref. (1) and Figures 3C–E]. Effects of IDO2 on T cell responses are indirect, as IDO2, like IDO1, is expressed in antigen presenting cells (APCs), but not in T cells [Figures 1A,B and refs. (1–3)]. To determine the relative contribution of IDO1 and IDO2 to the decreased T cell responses seen in IDO2 ko KRN.g7 mice, T cells and APCs from wt, IDO1 ko, IDO2 ko, and dko KRN.g7 mice were examined. To measure APC function, KRN T cells were stimulated with GPI peptide and I-A^g7^-expressing wt, IDO1 ko, IDO2 ko, or dko APCs. KRN T cells proliferate and upregulate the activation markers CD25 and CD69 in response to stimulation with peptide + wt APCs. No difference in T cell response was found when IDO1 ko, IDO2 ko, or dko APCs were used, demonstrating that the reduced T cell responses in IDO2 ko mice were not due to diminished APC function (Figure 4 and Supplementary Figure 4). To measure T cell subsets, KRN T cells were examined for expression of transcription factors and secretion of cytokines associated with Th subset differentiation. Percentages of CD4^+^ T cells expressing Th cell subset transcription factors (Tbet, Gata3, Rorγt, Bcl-6, Foxp3) (Figure 3A), the Tfh cytokine IL-21 (Figure 3B), and inflammatory cytokines IL-17a (Figure 3C and Supplementary Figure 3), TNFα (Figure 3D and Supplementary Figure 3), and IFNγ (Figure 3E and Supplementary Figure 3) were statistically indistinguishable in IDO1 ko and wt KRN.g7 mice, consistent with the lack of significant differences in numbers of autoantibody secreting cells and joint inflammation. Unexpectedly, dko KRN.g7 mice also had levels of differentiated autoreactive CD4^+^ T cells that were indistinguishable from wt KRN.g7 mice, despite their reduced arthritis and autoantibody production. Together, these data suggest that unlike the reduced autoreactive B cell response observed in IDO2 ko mice, which is strictly dependent on the loss of IDO2, the corresponding reduction of autoreactive T cell responses requires the retention of IDO1.

### IDO2 Does Not Influence B Cell Maturation or Response to *in vitro* Stimulation

**Figure 5:**
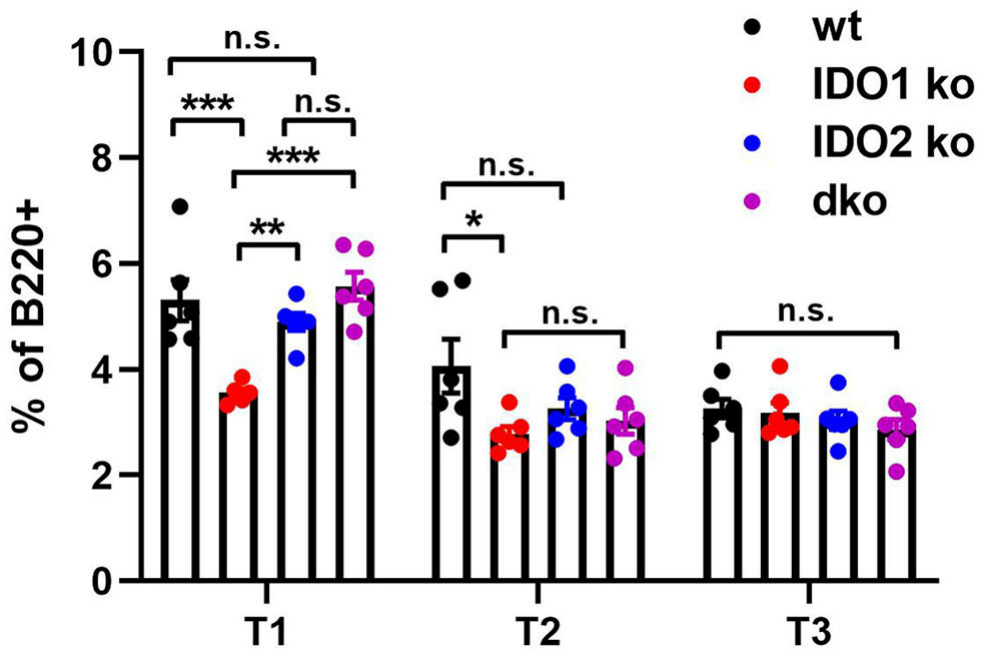
IDO2 does not affect maturation of B cells in the spleen. The frequency of transitional B cell subsets in wt, IDO1 ko, IDO2 ko, and dko spleens were measured by flow cytometry. Transitional B cell subpopulations were defined as T1 (B220^+^CD93^+^IgM^hi^CD23^−^), T2 (B220^+^CD93^+^IgM^hi^CD23^+^), and T3 (B220^+^CD93^+^IgM^low^CD23^+^). Symbols represent individual mice and histograms show the mean percentage out of total B cells ± SEM from 6 mice per genotype in 2 independent experiments. *P*-values were calculated by one way-ANOVA followed by comparison of means with Tukey's *post-hoc* multiple comparison correction. ^*^p < 0.05, ^**^p < 0.01, ^***^p < 0.001, n.s., not significant.

Diminished B cell activation and autoantibody secretion in arthritic IDO2 ko and dko, but not IDO1 ko, mice demonstrated an important role for IDO2 in driving autoreactive B cell responses. However, the requirement for IDO2 in normal B cell development and function is less clear. Previously, we demonstrated that IDO2 ko mice have normal populations of developing B cells in the bone marrow, mature follicular and marginal zone B cells in the spleen and B-1 and B-2 B cells in the peritoneal cavity (8). These subpopulations of B cells were also shown to be unaffected in IDO1 ko mice (42). Within the spleen, autoreactive B cells reside in a population of functionally immature transitional B cells and undergo a selection process prior to development into mature B cells (43–45). Transitional B cells are defined by expression of B220 and the early B lineage marker CD93 and can be divided into three subpopulations: T1 (CD93^+^IgM^hi^CD23^−^), T2 (CD93^+^IgM^hi^CD23^+^), and T3 (CD93^+^IgM^low^CD23^+^) (43). To determine if transitional B cell populations were affected by deletion of the IDO genes, spleens from IDO1 ko, IDO2 ko, and dko C57BL/6 mice were analyzed by flow cytometry (Figure 5 and Supplementary Figure 5). IDO1 ko mice had decreased percentages of T1 and T2, but not T3, B cells, compared to wt mice. In contrast, percentages of T1, T2, and T3 populations were unchanged in IDO2 ko, and dko mice, suggesting that IDO2 does not play a role in B cell maturation in the spleen (Figure 5).

**Figure 6:**
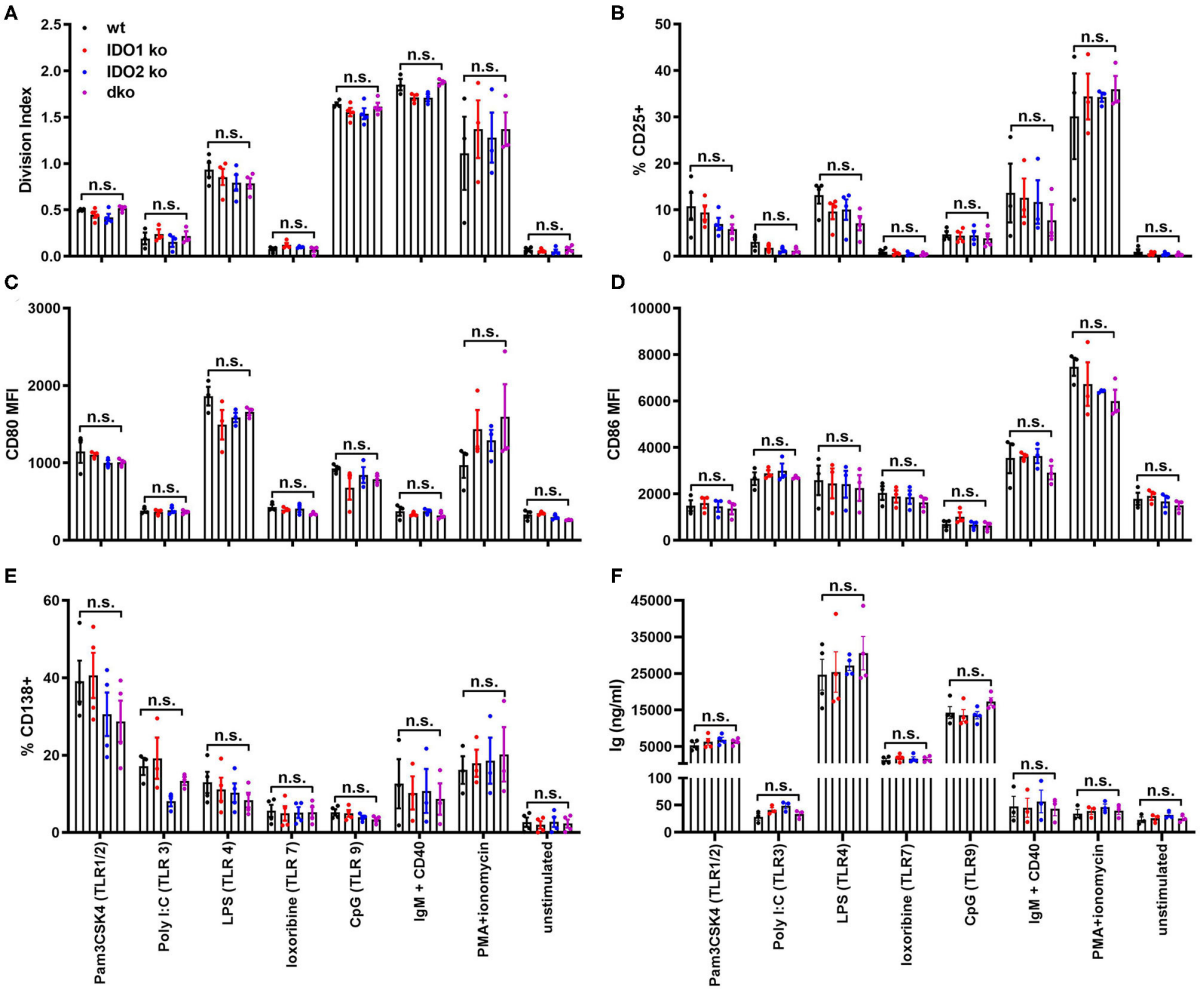
IDO2 ko B cells respond normally to stimulation with TLR ligands *in vitro*. Purified IDO1 ko, IDO2 ko, dko, or wt B cells were labeled with CFSE and stimulated *in vitro* with Pam3CSK4 (TLR1/2), Poly I:C (TLR3), LPS (TLR4), loxoribine (TLR7), CpG (TLR9), anti-IgM + anti-CD40, PMA + ionomycin, or media alone for 48 h. The cultured cells were then analyzed for **(A)** proliferation and the upregulation of activation markers **(B)** CD25, **(C)** CD80, **(D)** CD86, and **(E)** differentiation marker CD138 by flow cytometry. **(F)** The amount of Ig secreted into the supernatant was measured by ELISA. Symbols represent individual mice and histograms show mean ± SEM, pooled from a minimum of 3 independent experiments. *P*-values were calculated by one way-ANOVA followed by comparison of means with Tukey's *post-hoc* multiple comparison correction. n.s., not significant.

To better define the role IDO2 plays in normal B cell responses, we measured the ability of ko and wt B cells to proliferate, upregulate activation and costimulatory markers, and secrete antibody in response to stimulation *in vitro*. B cells can be activated by both antigen-specific and antigen non-specific stimuli like toll like receptors (TLRs), pattern recognition receptors expressed by immune cells that act as sensors to induce both innate and adaptive immune responses to infection and immunization. B cells express high levels of six TLRs, TLR1-4, TLR7, and TLR9 (46). To determine if IDO1 or IDO2 mediate B cell activation in response to TLR stimulation, purified B cells from wt, IDO1 ko, IDO2 ko, and dko C57BL/6 mice were cultured *in vitro* with the Toll-like receptor (TLR) ligands Pam3CSK4 (TLR1/2), Poly I:C (TLR3), LPS (TLR4), loxoribine (TLR7), and CpG (TLR9). Their responses were compared to stimulation with anti-IgM + anti-CD40 (B cell receptor + T cell help), PMA + ionomycin (positive control) or media alone (negative control). In agreement with published reports, B cells did not respond equally to the different modes of stimulation [Figure 6, Supplementary Figure 6, and ref. (46)]. Anti-IgM + anti-CD40 and PMA + ionomycin induced strong B cell proliferation, but not antibody secretion. The activation marker CD25, costimulatory molecules CD80 and CD86, and differentiation marker CD138 were strongly upregulated by PMA + ionomycin and all but CD86 were moderately upregulated by anti-IgM + anti-CD40. Stimulation with Pam3CSK4, LPS, and CpG induced B cells to proliferate, upregulate CD25, CD80, and CD86, and secrete antibody. Poly I:C induced modest proliferation, but not antibody secretion and loxoribine induced antibody secretion without proliferation. No differences were detected between IDO1 ko, IDO2 ko, dko, or wt B cells, suggesting that IDO2 does not affect the overall activation, proliferation, or differentiation of B cells to TLR stimulation *in vitro* (Figure 6 and Supplementary Figure 6).

### IDO2 Mediates *in vivo* B Cell Responses to Influenza Infection and Immunization With T Independent Type II Antigens

**Figure 7:**
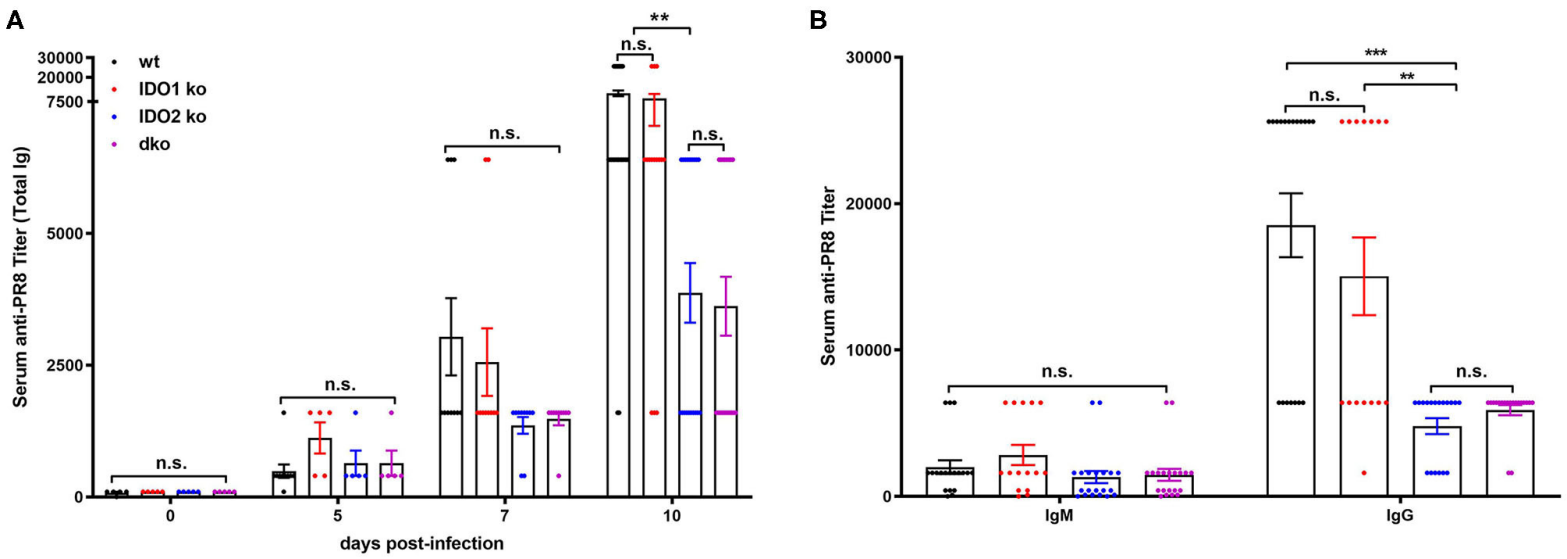
IDO2 deficient mice generate reduced antibody response to influenza. Wt, IDO1 ko, IDO2 ko, and dko C57BL/6 mice were infected with 200 TCID_50_ influenza virus (A/PR/8/34). **(A)** Titers of anti-PR8 Ig were measured on days 0, 5, 7, and 10 by ELISA. Symbols represent individual mice and histograms show mean serum anti-PR8 Total Ig titer ± SEM for a minimum of *n* = 10 mice per group. **(B)** Symbols represent individual mice and histograms show mean serum anti-PR8 IgM vs. IgG titers ± SEM on day 10 from *n* = 20 wt, *n* = 15 IDO1 ko, *n* = 20 IDO2 ko, and *n* = 20 dko B6 mice. Data are pooled from 4 independent experiments. *P*-values were calculated by one way-ANOVA followed by comparison of means with Tukey's *post-hoc* multiple comparison correction. ^**^p < 0.01, ^***^p < 0.0001, n.s., not significant.

Given the dichotomy between IDO2's critical role in mediating the autoreactive B cell response in the *in vivo* KRN model of autoimmune arthritis and the lack of effect on responses to *in vitro* stimuli, we used two well-characterized models to measure normal B cell responses *in vivo*, the PR8 influenza infection model (47) and (4-hydroxy-3-nitrophenyl) acetyl (NP) immunization model (48, 49). Previous studies of anti-influenza B cell responses have characterized a burst of low affinity IgM and high affinity IgG B cells in the 10 days following initial viral challenge (37). To determine if IDO2 affects these antibody response to flu infection, IDO1 ko, IDO2 ko, dko, and wt C57BL/6 mice were infected intranasally with A/PR/8/34 influenza virus (PR8) and titers of anti-PR8 Ig were measured in the serum on days 0, 5, 7, and 10. Anti-PR8 Ig titers were detectable at d5 and increased through d10 in wt B6 mice. No differences in anti-PR8 Ig titers were found in IDO1 ko mice. In contrast, anti-PR8 Ig titers were consistently lower in IDO2 ko and dko B6 mice (Figure 7A), signifying that IDO2 plays an important role in immune responses outside of the context of autoimmunity. These reduced anti-PR8 Ig titers were due to a reduction in both IgM and IgG anti-PR8 Ig (Figure 7B). Together, these data suggest an IDO1-independent role for IDO2 in the antibody response to influenza.

**Figure 8:**
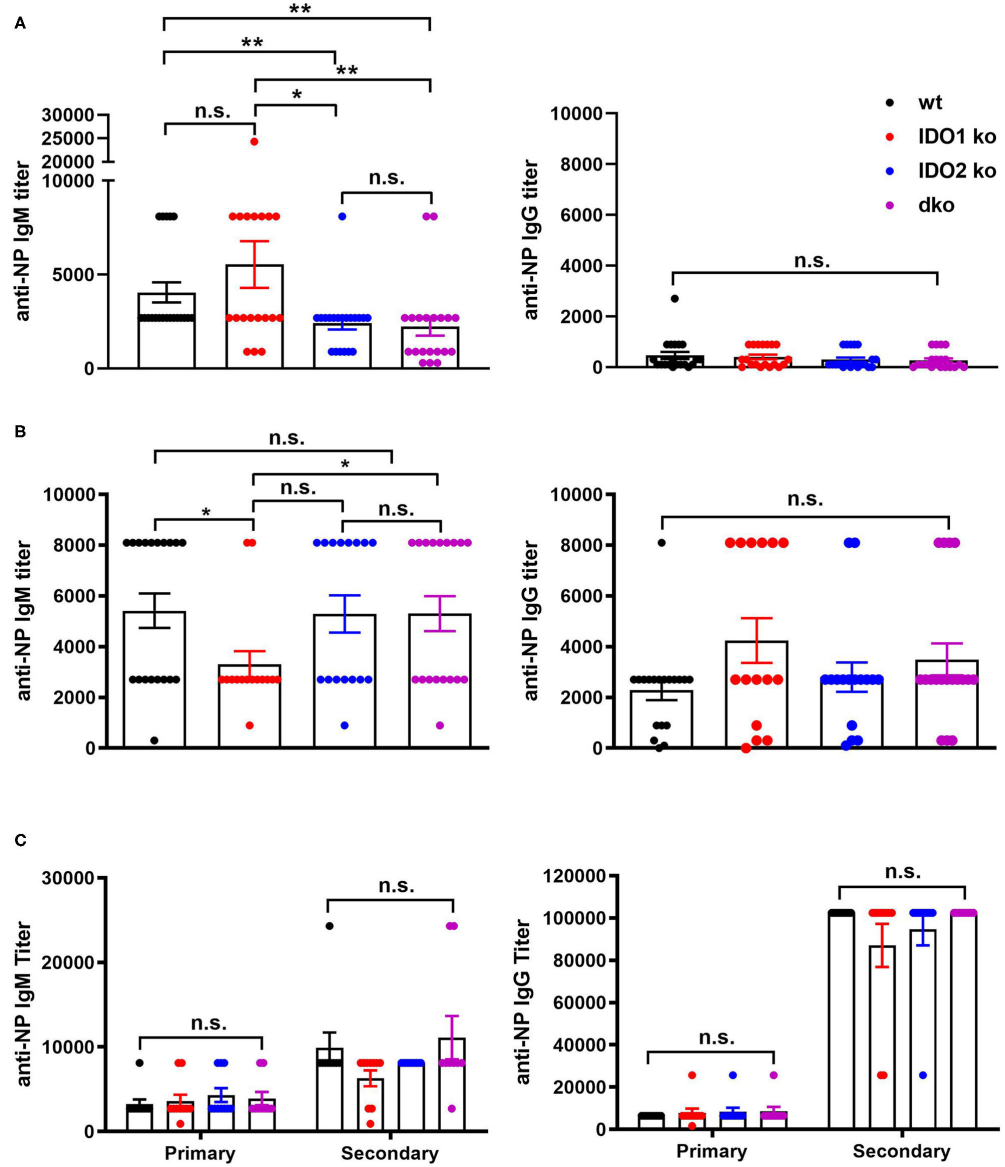
IDO2 ko and dko mice generate reduced antibody responses to T-independent type II, but not T-independent type I or T cell dependent responses. Wt, IDO1 ko, IDO2 ko, and dko C57BL/6 mice were immunized with **(A)** NP-Ficoll, **(B)** NP-LPS, or **(C)** NP-KLH and high affinity anti-NP titers measured by ELISA on day 7 (NP-Ficoll, NP-LPS) or day 10 after the primary and secondary responses (NP-KLH). Symbols represent individual mice and histograms show mean ± SEM of the reciprocal of serum anti-NP IgM and IgG titers from *n* = 20 mice per group for NP-Ficoll, pooled from 4 independent experiments; *n* ≥ 15 mice per group for NP-LPS, pooled from 3 independent experiments, and *n* = 10 mice per group for NP-KLH, pooled from 2 independent experiments. Individual symbols may overlap for mice with identical titers. *P*-values were calculated using a Mann-Whitney non-parametric test. ^*^p < 0.05, ^**^p < 0.01, n.s., not significant.

**Figure 9:**
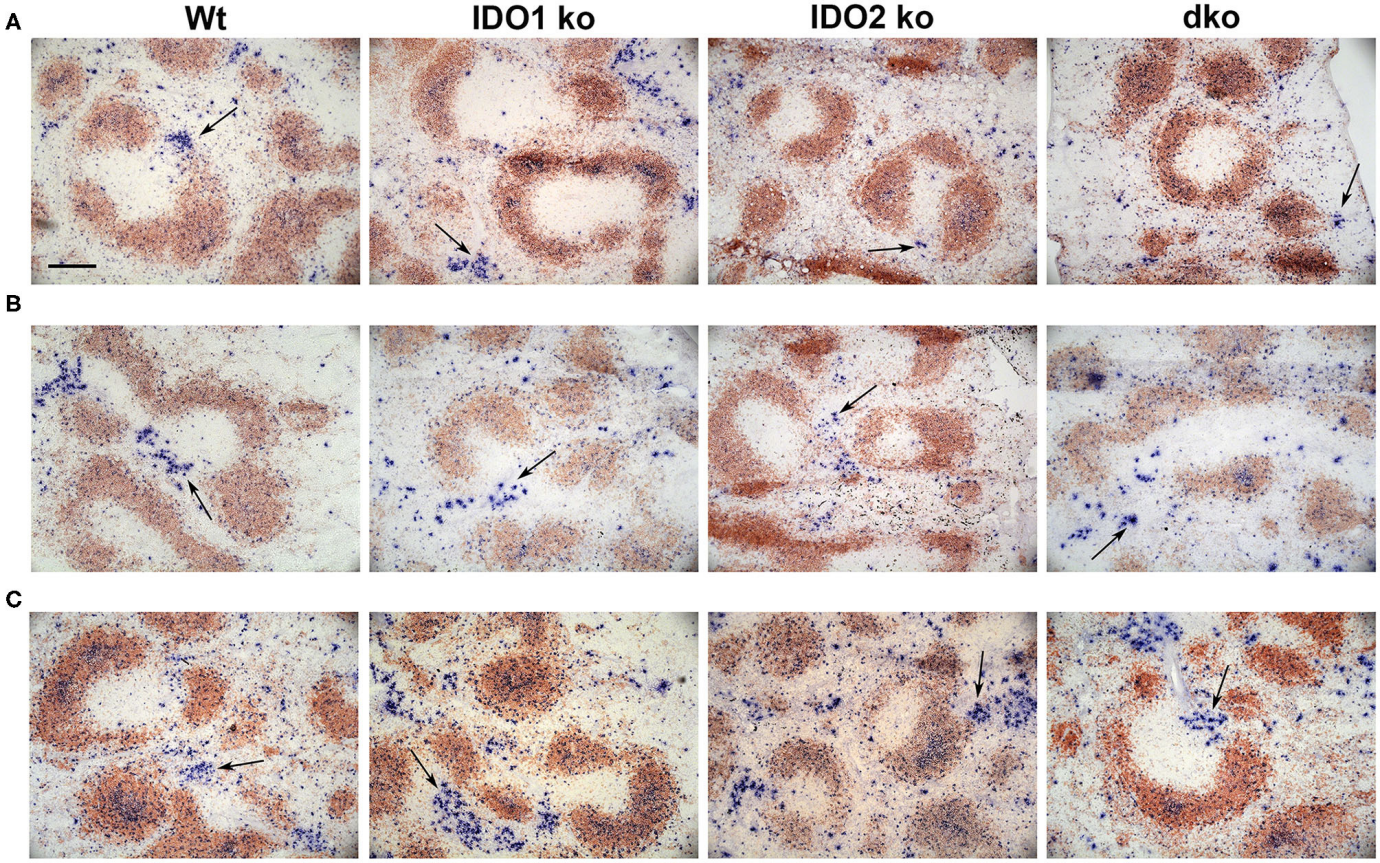
Localization of antibody secreting cells in immunized mice. Wt, IDO1 ko, IDO2 ko, and dko C57BL/6 mice were immunized with **(A)** NP-Ficoll, **(B)** NP-LPS, or **(C)** NP-KLH. Spleens were harvested on day 7 (NP-Ficoll and NP-LPS) or day 10 following primary immunization (NP-KLH). Sections were stained with B220 (red) and Igλ (blue). Representative images are shown from *n* = 3 mice per genotype from each immunogen. Antibody secreting cells (ASCs) are marked with arrows. Scale bar = 200μm.

The NP model can be used to distinguish factors that mediate B cell function in the context of both T cell dependent (TD) and independent (TI) responses (50). Our previous studies demonstrated normal primary and secondary antibody responses in IDO2 ko mice challenged with the TD antigen NP-KLH (1). IDO1 ko mice have also been shown to respond normally to the TD antigen NP-OVA, but to have an elevated response to the TI antigens NP-LPS and NP-Ficoll (42). To determine if IDO2 impacts B cell function differently in TD and TI responses, wt, IDO1 ko, IDO2 ko, and dko mice were immunized i.p. with NP-LPS (TI type I), and NP-Ficoll (TI type II) and compared to mice immunized with NP-KLH (TD). Serum anti-NP IgM and IgG titers were measured 7 days after immunization with NP-Ficoll or NP-LPS. IDO2 ko and dko mice generated reduced titers of IgM in response to the TI-type II antigen NP-Ficoll (Figure 8A), whereas their anti-NP titers in response to the TI type I antigen NP-LPS were similar to wt levels (Figure 8B). In contrast, IDO1 ko mice trended toward higher IgM anti-NP titers in response to NP-Ficoll and IgG anti-NP titers to NP-LPS, as had been previously reported [Figures 8A,B and ref. (42)]. Consistent with our previously published work, wt, IDO1, and IDO2 ko mice generated robust primary and secondary antibody responses to immunization with the TD antigen NP-KLH [Figure 8C and ref. (1)]. No differences were detected in either the IgM or IgG anti-NP titers. Dko mice also generated robust anti-NP titers following primary and secondary immunization, indicating that there was no compensatory effect of IDO1 in the IDO2 ko in response to NP-KLH (Figure 8C). The anti-NP response can be characterized histologically by the presence of Igλ expressing antibody secreting cells (ASCs) localized in the bridging channels and red pulp (51). These Igλ ASCs were prevalent in wt and IDO1 ko mice immunized with NP-Ficoll, but were reduced in IDO2 ko and dko mice, consistent with their reduced serum anti-NP titers (Figure 9). No differences in Igλ ASCs were found in wt, IDO1 ko, IDO2 ko, or dko mice immunized with NP-LPS or NP-KLH (Figure 9). Together, these data demonstrate that IDO2 does not affect all B cell responses, but specifically mediates antibody responses to influenza and T cell independent type II antigens.

## Discussion

IDO1 and IDO2 are closely linked, homologous genes. Despite their genetic and structural similarities, clear functional differences between IDO1 and IDO2 have been observed, both in their roles as potential tryptophan catabolic enzymes and in their connection to immune function. Teasing apart the individual contributions of IDO1 and IDO2 in immunity has been a challenge for the field, a problem conflated with the use of mouse model deletion systems, where the effect of deletion of one IDO gene may have unknown consequences on the expression and potential compensatory role of the other. Exaggerated inflammatory responses in IDO1 ko mice and reduced autoimmune responses in IDO2 ko mice have been used to ascribe immune regulatory vs. proinflammatory roles for IDO1 and IDO2, respectively. Here, we demonstrate that IDO1 ko mice have reduced levels of IDO2 and IDO2 ko mice have increased levels of IDO1 in certain cells/tissues. This suggests that care must be taken in interpreting results from studies of immunity conducted using single ko mice, as the effect could be due to altered expression of the other IDO protein. Initial studies using mice genetically deficient in both IDO1 and IDO2 showed that for at least one of these responses (elevated IL-10 production in IDO1 deficient macrophages), the phenotype was not seen when IDO2 was also deleted (34). This highlights the need to use both IDO1 and IDO2 single and double ko mice to distinguish the individual impact of IDO1 and IDO2 in immune responses. In this study, we used both single and double ko's to definitively determine the role of each enzyme in models of B cell-mediated autoimmunity and inflammation.

Our previous work demonstrated reduced autoreactive B and T cell responses and attenuated disease in IDO2 ko, but not IDO1 ko, KRN.g7 mice, suggesting that IDO2 was an important mediator of both B and T cell responses driving autoimmunity (1). Here, we find that autoantibody levels and arthritis were also reduced in IDO1/IDO2 dko mice, confirming that IDO2 mediates autoreactive B cell responses in an IDO1-independent manner. However, autoreactive T cell responses were not reduced in dko mice, indicating that the autoreactive T cell defect in IDO2 ko mice was indirectly associated with the expression of IDO1. This is consistent with enhanced T cell responses reported when IDO1 was deleted in other models, including induced models of cancer (19, 52) and T cell-mediated autoimmunity (24, 53). In addition to having reduced autoreactive B cell responses, IDO2 ko mice generated reduced serum antibody titers in response to influenza infection and immunization with the T cell independent type II antigen, NP-Ficoll. These reduced B cell responses were also observed in dko mice, demonstrating a direct effect for IDO2 in normal B cell responses. These reduced antibody responses were not due to differences in B cell maturation, as B cell development in the bone marrow and maturation in the spleen were normal in IDO2 ko and dko mice. T-independent type I (NP-LPS) and T-dependent (NP-KLH) responses were not affected in IDO2 ko or dko mice. Similar to previous studies, IDO1 ko mice showed a trend toward elevated antibody titers in response to immunization with T cell independent antigens (42). This correlated with a decrease in T1 and T2 B cells in our study; however, T1 and T2 B cell populations were not found to be reduced in another report (42). Prior to this study, antibody responses to influenza infection had not been evaluated in IDO1 ko mice, though T cell responses were shown to be elevated following infection (54).

While IDO2 was clearly necessary for full B cell responsiveness in the *in vivo* models of NP-Ficoll immunization, influenza, and autoimmunity, *in vitro* tests of B cell function did not show differences between IDO1 ko, IDO2 ko, or dko responses. The difficulty of replicating IDO2's role in these *in vitro* systems suggests that the function of IDO2 is not solely intrinsic to B cells, and it may require feedback or interactions from other components of the immune system to exert it's proinflammatory effects. Alternatively, since the immune pathways modulated by IDO2 are not known, it may simply be that stimuli applied *in vitro*, such as TLR agonists or B cell receptor stimuli, are bypassing the pathways in which IDO2 plays a role. Either way, it is important that the immune mechanisms associated with IDO2 be assessed in a full immune context and serves as a reminder of the limitations of *in vitro* studies when assessing gene function.

Our work shows a clear connection between IDO2 and B cell-mediated autoimmunity, however, the molecular mechanism by which IDO2 directs autoimmune and other inflammatory B cell responses is not known. In addition, the role of the other tryptophan catabolizing enzymes, IDO1 and TDO, in B cell processes are little studied. Historically, the IDO enzymes have been linked to immune modulation through their ability to both deplete the local tryptophan concentration and generate immunomodulatory tryptophan catabolites. The amino acid sensing enzymes GCN2 and mTOR may both play a part in detecting IDO-mediated tryptophan depletion, signaling cell cycle arrest as well promoting the differentiation of CD4^+^ T cells into regulatory T cells (55–57). In addition, tryptophan catabolites have been shown to induce both regulatory T and B cells through activation of the aryl hydrocarbon receptor (AhR) (58–60). Enhanced AhR activity is associated with disease in autoimmune patients and preclinical models of disease, and AhR agonists have been shown to inhibit inflammation in models of inflammatory bowel disease, systemic lupus erythematosus, and rheumatoid arthritis (61–63). The three tryptophan catabolizing enzymes (IDO1, IDO2, and TDO) can each initiate the kynurenine pathway, and as such, could modulate inflammatory immune function through an AhR-dependent mechanism (64). However, unlike IDO1 and TDO which have robust tryptophan catabolizing activity, IDO2's enzymatic activity is extremely weak and IDO2 deletion does not affect circulating levels of tryptophan catabolites (1, 8, 41, 65). This has led us and others to propose that IDO2's main function is not through tryptophan catabolism, but through an as yet unidentified pathway. In support of this, IDO1 has also been shown to have signaling roles outside of its tryptophan catabolizing function (66–68). Ongoing work to determine the downstream effector pathway mediated by IDO2 will be important to understand the differential effects of IDO1 and IDO2 loss on the inflammatory B cell responses examined in the current study.

In summary, IDO1 and IDO2 appear to have contrasting roles in immunity, with IDO1 mediating T cell suppressive effects and IDO2 working directly in B cells as a proinflammatory mediator of autoimmune processes. IDO2's immunological effects are not limited to autoimmune model systems but influence some aspects of normal B cell function as well. IDO2 seems to be the dominant player in many B cell-mediated immune responses, with dko mice generally phenocopying the IDO2 ko in models of autoimmune arthritis, influenza, and NP-immunization. Finally, findings from studies using IDO1 or IDO2 single ko mice should be confirmed in double knockout mice, as deletion of one IDO gene affects the expression of the counteracting gene.

## Data Availability Statement

All datasets generated for this study are included in the article/Supplementary Material.

## Ethics Statement

Studies were performed in accordance with National Institutes of Health and Association for Assessment and Accreditation of Laboratory Animal Care guidelines with approval from the LIMR Institutional Animal Care and Use Committee.

## Author Contributions

LM designed and performed the immunization, flow cytometry and *in vitro* studies performed the statistical analysis, and assisted in the writing of the manuscript. JD performed the influenza experiments. JM performed the immunohistochemistry studies. W-DP performed the Western blotting studies. AC provided the PR8 virus. PM provided the IDO1/IDO2 dko mice. AC, PM, AM, and GP contributed to the conception and interpretation of the study. LM-N designed and performed the arthritis and influenza experiments, directed the work, and prepared the manuscript. All authors contributed to the manuscript revision, read, and approved the submitted version.

## Conflict of Interest

GP declares conflicts of interest as a compensated scientific advisor for Kyowa Kirin Pharmaceutical Development, Inc. and KYN Therapeutics, Inc., which are engaged in drug discovery and development in IDO pathways. JD, AM, and GP are inventors on issued patents claiming structure of matter and therapeutic uses of IDO1 inhibitors. GP, LM, and LM-N are inventors on patents that claim IDO2 nucleic acid sequences and IDO2 antibodies. The remaining authors declare that the research was conducted in the absence of any commercial or financial relationships that could be construed as a potential conflict of interest.
