# Supplementary figures and images for: Differential Roles of IDO1 and IDO2 in T and B Cell Inflammatory Immune Responses
Source: Front Immunol. 2020 Aug 18;11:1861. doi: 10.3389/fimmu.2020.01861 (PMC7461966; doi:10.3389/fimmu.2020.01861)

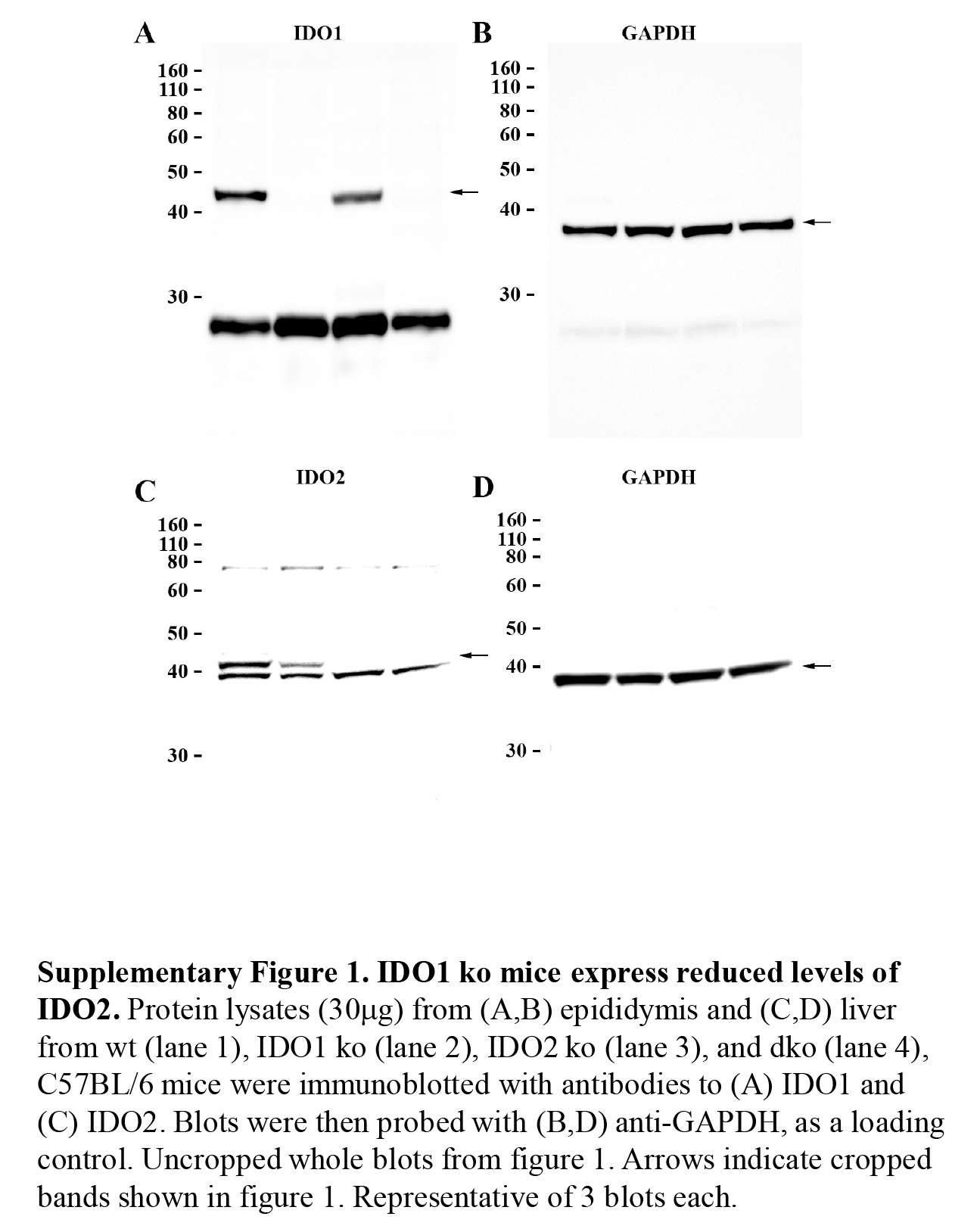

Supplement: Supplementary file 1 [file Image_1.TIF]

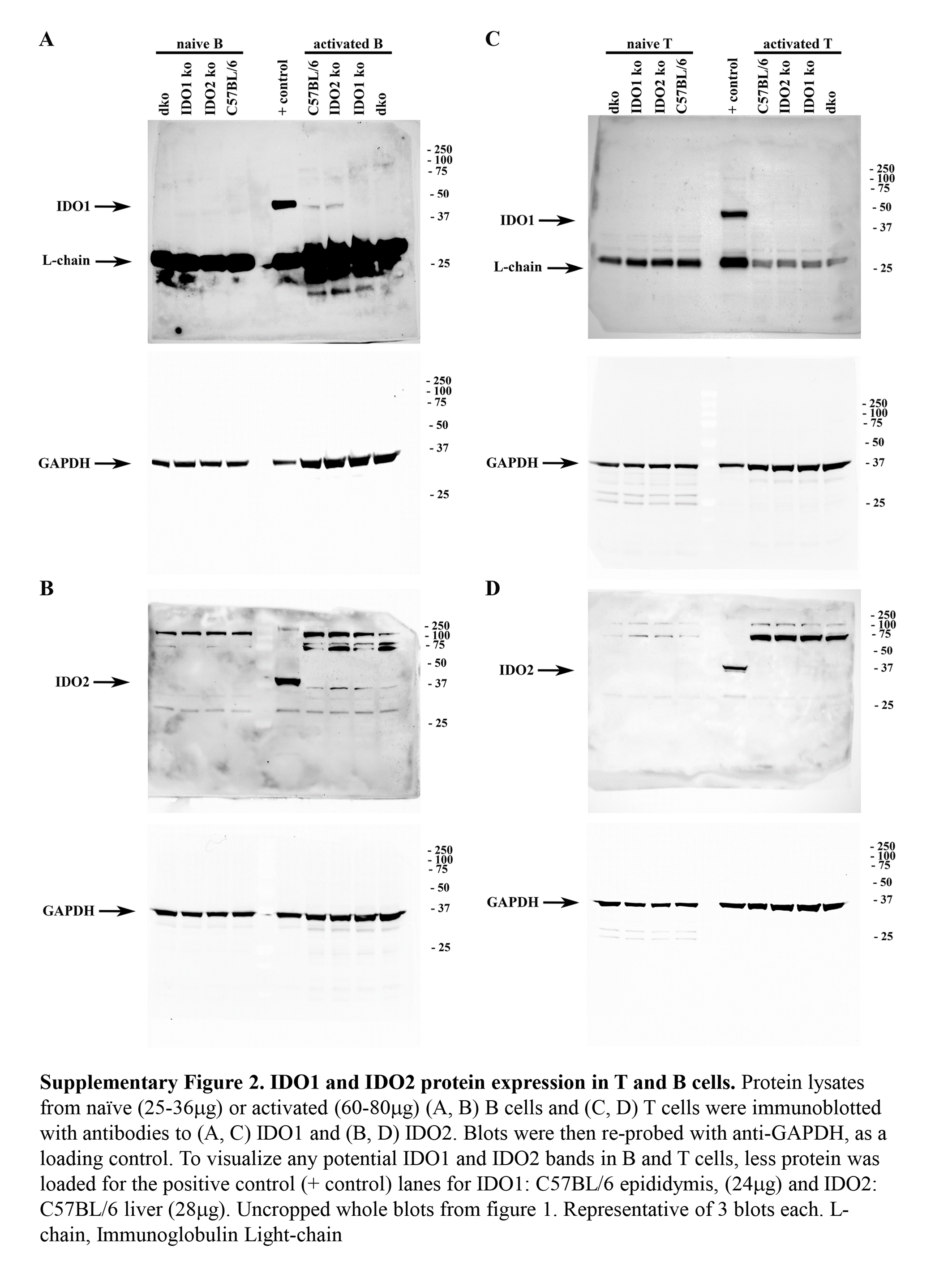

Supplement: Supplementary file 2 [file Image_2.TIF]

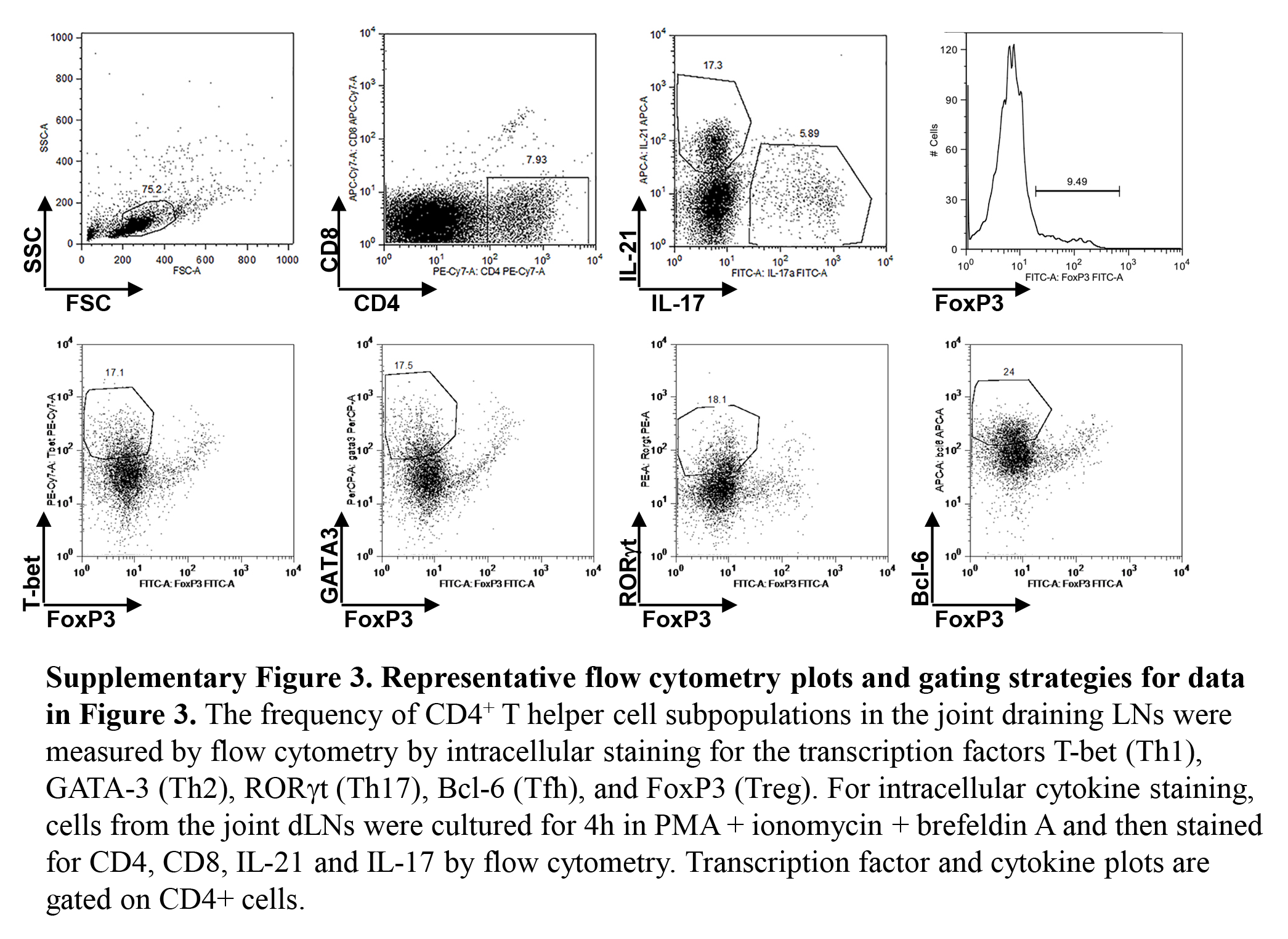

Supplement: Supplementary file 3 [file Image_3.TIF]

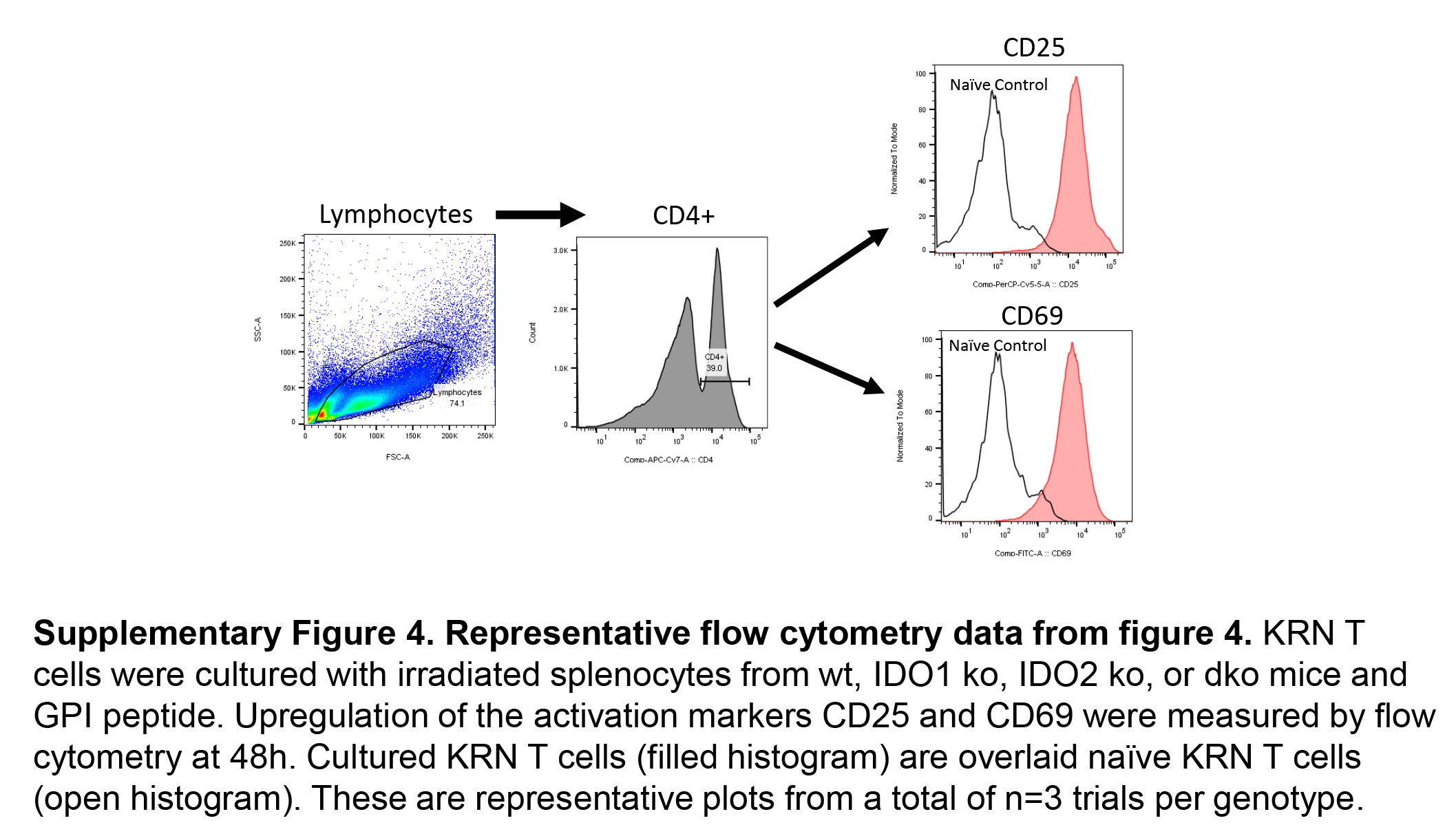

Supplement: Supplementary file 4 [file Image_4.TIF]

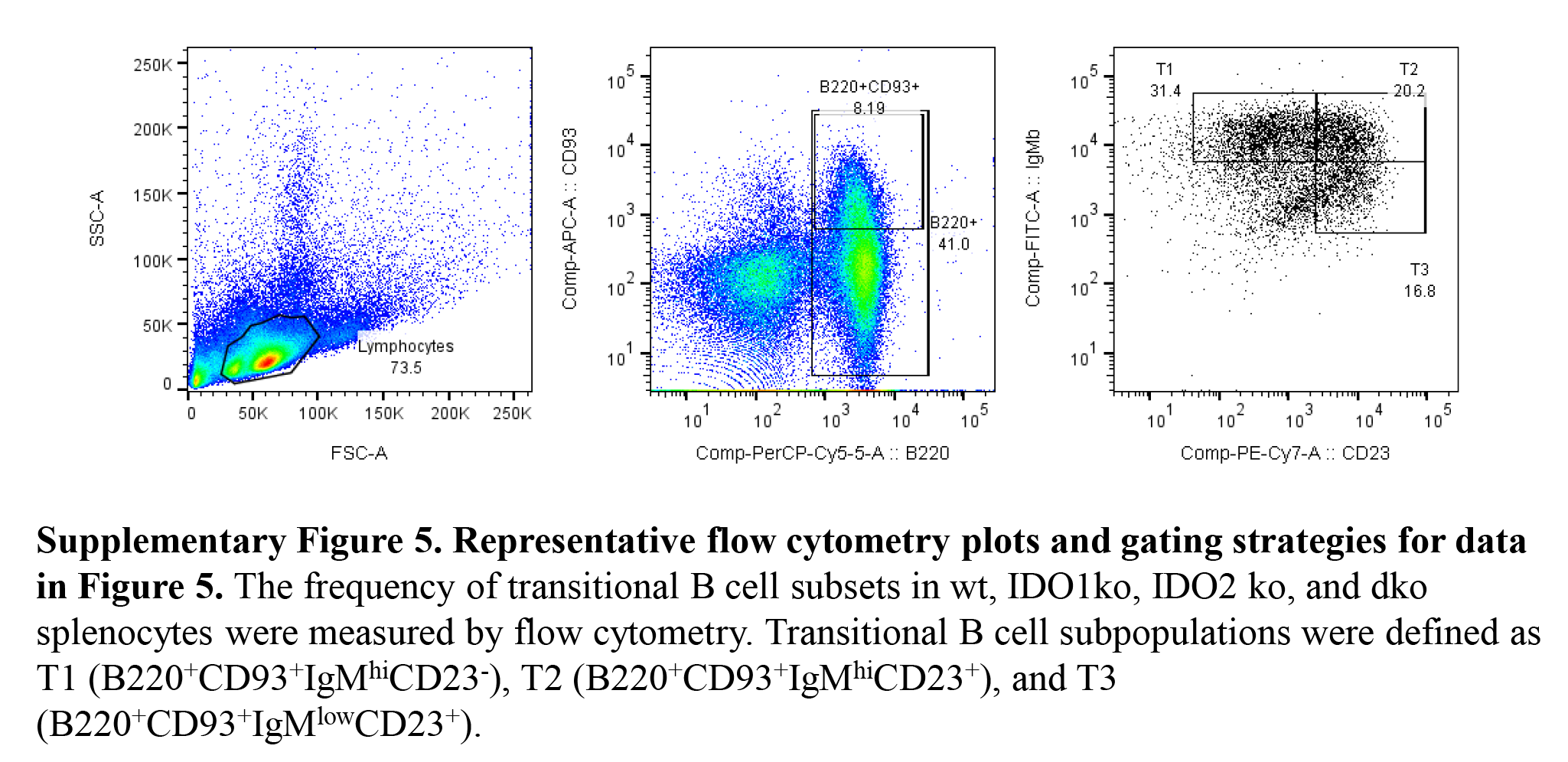

Supplement: Supplementary file 5 [file Image_5.TIF]

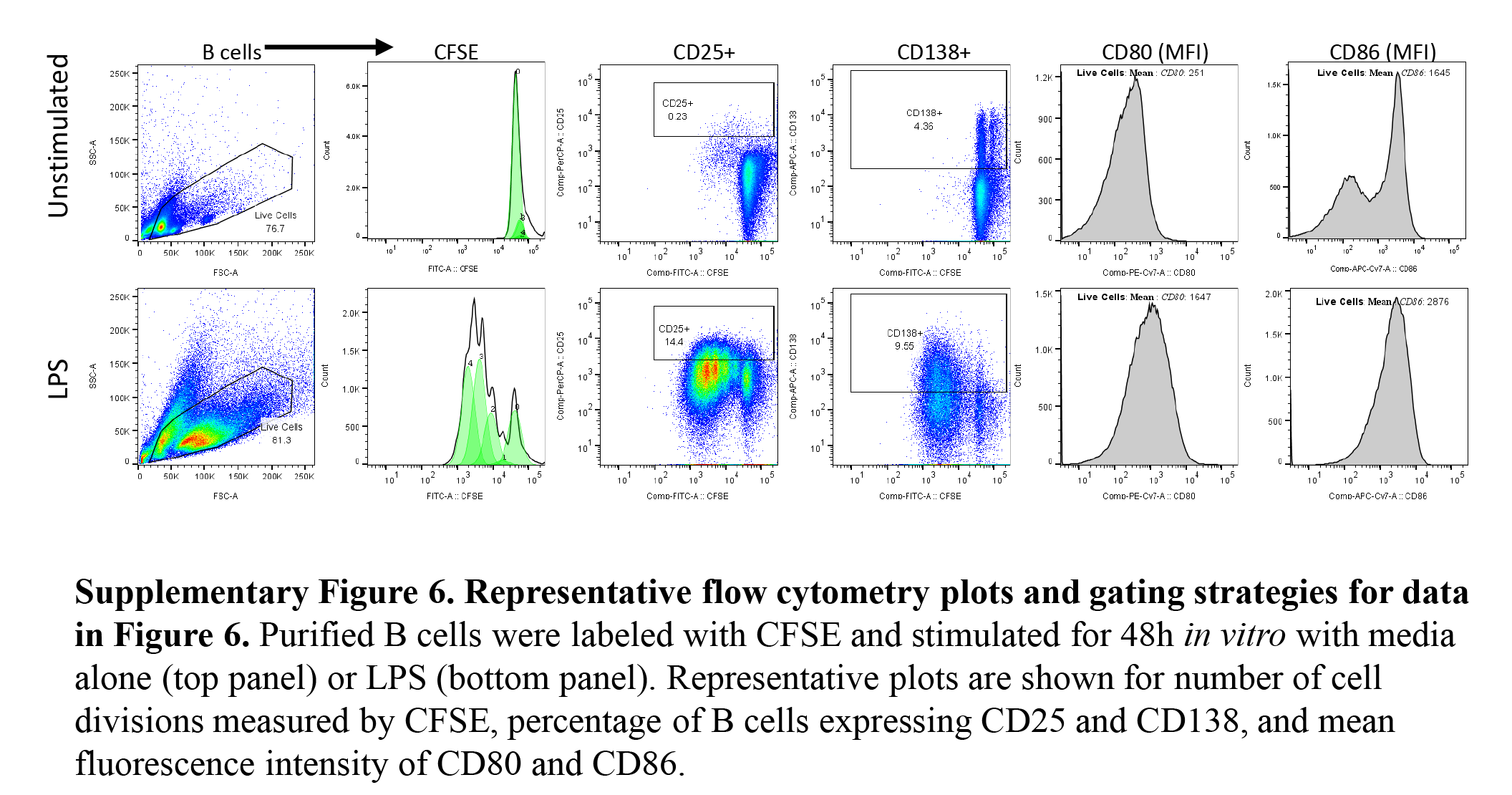

Supplement: Supplementary file 6 [file Image_6.TIF]
